# Glaukomversorgung in Deutschland – Ergebnisse einer Mitgliederumfrage von DOG und BVA – Teil 1: Diagnostik 

**DOI:** 10.1007/s00347-021-01352-1

**Published:** 2021-03-17

**Authors:** Christian Wolfram, Alexander K. Schuster

**Affiliations:** 1 Augenklinik und Poliklinik, Universitätsklinikum Hamburg-Eppendorf, Martinistr. 52, 20246 Hamburg, Deutschland; 2 Augenklinik und Poliklinik, Universitätsmedizin der Johannes Gutenberg-Universität Mainz, Mainz, Deutschland

**Keywords:** Versorgungspraxis, OCT, Spaltlampe, Gonioskopie, Übereinstimmung, Care practice, OCT, Slit-lamp, Gonioscopy, Agreement

## Abstract

**Hintergrund:**

Die Möglichkeiten in der Glaukomversorgung sind in den letzten Jahren immer vielfältiger geworden. Unter den Mitgliedern der Deutschen Ophthalmologischen Gesellschaft (DOG) und des Berufsverbands der Augenärzte Deutschlands (BVA) wurde eine anonymisierte Befragung durchgeführt, um zu erfassen, welche Behandlungswege in der Versorgung von Glaukompatienten gewählt werden, welche diagnostischen Parameter den Augenärztinnen und Augenärzten wichtig sind und welche Rolle Behandlungsleitlinien in der Alltagspraxis zukommt.

**Ziel der Arbeit:**

Meinungsbild unter der deutschen Augenärzteschaft über die aktuelle Glaukomversorgung, zentrale diagnostische Parameter und ihre Anwendung in der Alltagspraxis.

**Material und Methoden:**

Die Befragung wurde durch einen Online-Fragebogen mit insgesamt 26 Fragen (107 Items) zur Versorgungspraxis in der Glaukomdiagnostik und der Glaukomtherapie durchgeführt. Vollständig ausgefüllte Fragebögen lagen von 1361 Personen vor.

**Ergebnisse:**

Die Papillenbeurteilung an der Spaltlampe hat für Augenärzte weiterhin den höchsten diagnostischen Stellenwert. Auch der Rolle der optischen Kohärenztomographie (OCT) kommt eine sehr hohe diagnostische Bewertung zu. Bei der Interpretation verschiedener diagnostischer Parameter ergibt sich eine höhere Sicherheit für papillennahe Parameter unter den Befragten. Eine leitliniengemäße Versorgung wird nach Selbstauskunft der Augenärzte weitgehend betrieben. Etwa zwei Drittel der Befragten führen im ersten Behandlungsjahr zwei oder mehr Gesichtsfelduntersuchungen durch und ebenso eine strukturelle Papillendarstellung einmal im Jahr. Die Rolle der Gonioskopie wird kontrovers gesehen und nicht einheitlich intensiv praktiziert.

**Diskussion:**

Die Diagnosestellung beim Glaukom ist eine große klinische Herausforderung. Die verschiedenen diagnostischen Parameter haben einen unterschiedlich hohen Wert für Augenärzte. Morphometrische Verfahren haben eine sehr hohe Bedeutung gewonnen und ermöglichen eine assistierte, jedoch keine automatisierte Diagnostik. Mehr Behandlungsleitlinien und Standards bei der Glaukomversorgung werden gewünscht und sollten in Aus- und Weiterbildung und in den Behandlungsalltag implementiert sein.

## Abstract (en)

**Background:**

Glaucoma care has extended enormously in recent years. We performed a survey among the members of the German Ophthalmological Society (DOG) and the Professional Association of Ophthalmologists in Germany (BVA) to explore treatment patterns, the importance of different diagnostic parameters as well as treatment guidelines in the daily practice of glaucoma care.

**Objective:**

Assessment of the current practice of glaucoma care among German ophthalmologists, evaluation of key diagnostic parameters and their use in daily practice.

**Material and methods:**

Our survey was conducted using an online questionnaire that consisted of 26 questions (107 items) about the treatment practice for the diagnosis and treatment of glaucoma. Complete responses were available from 1361 participants.

**Results:**

Slit-lamp examination still remains the most important tool for diagnosis in glaucoma. A very high ranking was also achieved for optical coherence tomography (OCT) parameters. With respect to clinical interpretation and diagnostic certainty parameters analyzing the optic disc were ranked higher than others. Guideline-oriented care is being practiced by the vast majority of ophthalmologists according to self-reported results—two thirds perform perimetry two or more times and a structural analysis once in the first year after diagnosis. The importance of gonioscopy is controversial among the survey participants and is therefore being practiced with different intensity.

**Discussion:**

The diagnosis of glaucoma is challenging. Different diagnostic parameters are of different values to the ophthalmologists. Morphometric tools have achieved a highly important role for the diagnosis of glaucoma and enable assisted, yet not automated diagnosis. Treatment guidelines and standards are requested and should be implemented in education and care practice.

## Hintergrund und Fragestellung

Das Glaukom zählt zu den häufigsten Augenerkrankungen mit einer Prävalenz von 0,9–2,4 % in der erwachsenen Bevölkerung [12], entsprechend einer Häufigkeit von etwa 1 Mio. betroffener Patienten und einer weiteren Million Verdachtsfälle in Deutschland [23]. Es ist nach der Makuladegeneration der häufigste Grund für Sehbehinderung und Blindheit in Deutschland [5]. Trotz dieser hohen Relevanz als Gesundheitsproblem erscheint die Glaukomversorgung sehr heterogen und für viele unübersichtlich. Die diagnostischen und therapeutischen Möglichkeiten der Behandlung von Glaukomen haben sich in den letzten Jahren stetig weiterentwickelt. Insbesondere die Möglichkeiten zur Darstellung und Ausmessung des Sehnervenkopfes haben die Glaukomdiagnostik erweitert. Daher stellen sich Fragen, welche diagnostischen Mittel in der Breitenversorgung am ehesten Anwendung finden und welchen Stellenwert die behandelnden Ophthalmologen diesen beimessen. Ist die Beurteilung des Sehnervenkopfes an der Spaltlampe noch „state of the art“? Welche Rolle kommt der Diagnostik durch die Optische Kohärenztomographie (OCT) im Alltag zu? In welchem Umfang wird die Gonioskopie praktiziert?

Anhand einer Umfrage unter Augenärztinnen und Augenärzten in Deutschland wurde dazu die Praxis der Glaukomdiagnostik in der Alltagsversorgung hinterfragt.

Neben der Bedeutung und Anwendung verschiedener diagnostischer Instrumente und Parameter ging es auch um die Rolle von Behandlungsleitlinien und den Einfluss von individuellen Gesundheitsleistungen (IGeL) in der Diagnostik.

## Studiendesign und Untersuchungsmethoden

Im Mai 2020 wurde eine anonymisierte Online-Umfrage zur Glaukomversorgung unter den Mitgliedern der Deutschen Ophthalmologischen Gesellschaft (DOG) sowie des Berufsverbandes der Augenärzte Deutschlands (BVA) durchgeführt. Dazu wurden insgesamt 9702 Augenärzte mittels E‑Mail zur Umfrage eingeladen und um die Teilnahme, die über einen Link zugänglich war, gebeten. In einer weiteren E‑Mail wurde einmalig an die Teilnahme erinnert. Der Fragebogen bestand aus insgesamt 26 Fragen (107 Items) zur Versorgungspraxis in der Glaukomdiagnostik und der Glaukomtherapie sowie zur Einschätzung der Rolle von Leitlinien. Die Befragten hatten darüber hinaus auch die Möglichkeit, über offene Kommentarfelder Anmerkungen zu ihrer Sicht der Glaukomversorgung zu machen. Im Vorfeld der Umfrage wurden 3 unabhängige Glaukomexperten hinzugezogen, um die Themenfelder und den Umfang der Befragung zu konsentieren.

Insgesamt nahmen 1571 Personen an der Befragung teil, wobei der Fragebogen von 1361 (86,6 %) Personen vollständig ausgefüllt wurde, entsprechend einer Rücklaufquote von 16,2 % bzw. 14,0 % im Verhältnis zur Anzahl der verschickten Einladungen. Alle Bundesländer waren in der Umfrage vertreten – vom Saarland (*N* = 14) bis Nordrhein-Westfalen (*N* = 299).

Die Ergebnisse wurden nach absoluten und relativen Häufigkeiten der Antworten analysiert. Weiterhin wurde eine Stratifizierung nach Subgruppen (Geschlecht, Alter, Berufserfahrung, beruflichem Status, Versorgungssektor sowie nach chirurgischer oder konservativer Tätigkeit) durchgeführt, und die Subgruppen wurden miteinander verglichen. Da es sich um eine rein explorative Untersuchung handelt, wurde auf eine Prüfung auf statistische Signifikanzunterschiede zwischen Subgruppen verzichtet.

Zur einfacheren Lesbarkeit wurde im Manuskript stets nur ein Geschlecht genannt, es sind jedoch durchgängig beide Geschlechter hiermit gemeint. Da die Ergebnisse der gesamten Befragung zur Glaukomversorgung insgesamt zu umfangreich sind, werden in dieser Publikation nur die Ergebnisse zur Glaukomdiagnostik vorgestellt. Die Resultate zur Glaukomtherapie werden in einer separaten Arbeit veröffentlicht.

## Ergebnisse

Unter den Umfrageteilnehmern waren 52,8 % weiblichen und 46,0 % männlichen Geschlechts; 80,4 % waren Fachärzte, 6,7 % Weiterbildungsassistenten und 8,2 % Augenärzte in leitender Position (Chef- oder Oberärzte); 86,3 % waren in der Niederlassung tätig, davon 40,0 % in einer Einzelpraxis, 43,1 % in einer Gemeinschaftspraxis und 16,9 % in einem medizinischen Versorgungszentrum (MVZ); 57,7 % der Umfrageteilnehmer gaben an selbstständig zu sein gegenüber 41,9 % in einem Anstellungsverhältnis; 13,4 % der Befragten arbeiteten in einer Augenklink, davon jeweils etwa zur Hälfte in Universitätsaugenkliniken (51,1 %) und in nichtuniversitären Augenkliniken (48,9 %); 26,4 % verfügten über eine Berufserfahrung von 30 oder mehr Jahren, 15,7 % von weniger als 10 Jahren.

## Herausforderung Diagnose

**Figure 1:**
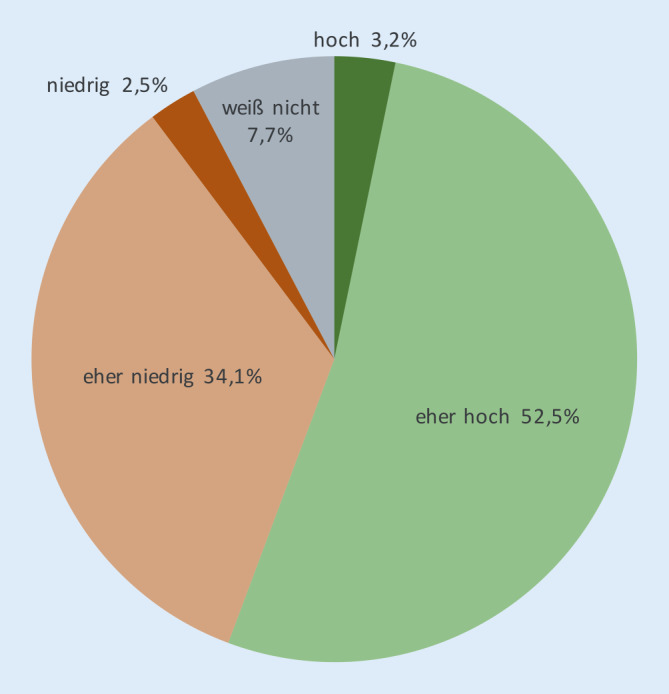

Die Diagnosestellung beim Glaukom ist eine besondere klinische Herausforderung, bei der sich unterschiedliche Auffassungen der Augenärzte darstellen. So ergibt die Frage nach dem Grad der Übereinstimmung zwischen verschiedenen Augenärzten bei der Erstdiagnose eines behandlungsbedürftigen Glaukoms eine heterogene Einschätzung unter den befragten Augenärzten: Knapp über die Hälfe nehmen eine hohe oder eher hohe Übereinstimmung an, während über ein Drittel der Befragten diese für niedrig oder eher niedrig halten (Abb. 1).

Dabei ergeben sich kleine Unterschiede zwischen den Subgruppen: Frauen nehmen etwas mehr Übereinstimmung an als Männer, Berufserfahrene mehr als Berufsanfänger und konservativ tätige mehr als chirurgisch tätige Ophthalmologen. Chef- und Oberärzte erweisen sich als besonders skeptisch: Hier gehen 60,4 % von einer eher niedrigen bis niedrigen Übereinstimmung aus.

## Bewertung einzelner diagnostischer Parameter

**Figure 2:**
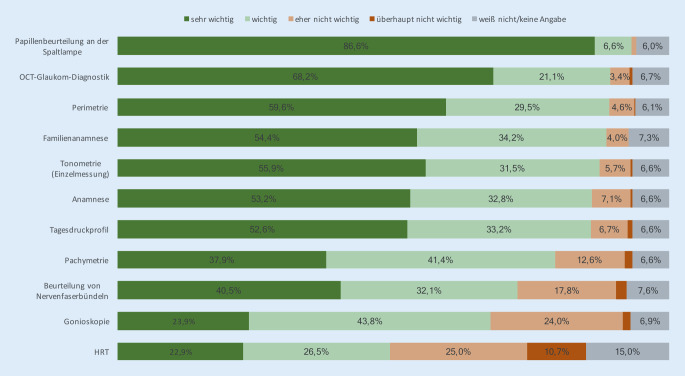

Gefragt nach der Bedeutung verschiedener diagnostischer Parameter, fällt auf, dass der Beurteilung der Papille an der Spaltlampe weiterhin eine zentrale Rolle zugemessen wird. Auch der Glaukomdiagnostik mittels optischer Kohärenztomographie (OCT) wird eine wichtige Bedeutung eingeräumt, die nicht nur gegenüber dem Heidelberg Retina Tomograph (HRT, Heidelberg Engineering GmbH, Heidelberg, Deutschland), sondern auch gegenüber den anderen glaukomdiagnostischen Parametern deutlich heraussticht (Abb. 2).

Die verschiedenen Subgruppen geben in der Beurteilung der verschiedenen Glaukomparameter jeweils eine weitgehend ähnliche Wertigkeit an. Leichte Unterschiede sind jedoch feststellbar mit einer höheren Gewichtung von Tagestensioprofilen und Gesichtsfeldmessungen in Augenkliniken gegenüber niedergelassenen Ophthalmologen, die wiederum der Tonometrie als Einzelmessung ein etwas höheres Gewicht einräumen. Ebenso ist eine leicht höhere Einschätzung feststellbar durch Augenärztinnen für die Rolle der Anamnese und Familienanamnese im Vergleich mit männlichen Kollegen.

## Sicherheit in der Interpretation verschiedener diagnostischer Parameter

**Figure 3:**
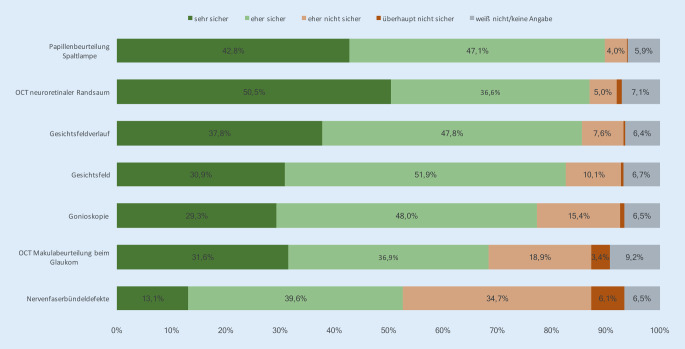

In Bezug auf die persönliche Sicherheit für die Interpretation der verschiedenen Glaukomparameter wird ebenfalls für die Papillenbeurteilung an der Spaltlampe von den Befragten die höchste Einschätzung gewählt. Bei der OCT-Diagnostik überwiegt deutlich die Beurteilung des neuroretinalen Randsaums (peripapilläre retinale Nervenfaserschichtdicke) gegenüber der Makuladiagnostik. Unsicherheiten wurden für die Beurteilung von Nervenfaserbündeldefekten angegeben. Vereinfacht lässt sich somit feststellen, dass mehr Sicherheit für die *papillennahe* Diagnostik festgestellt wird, während bei Diagnoseparametern *außerhalb* der Papille eher Unsicherheiten benannt werden (Abb. 3).

Im Vergleich zwischen den Gruppen ergibt sich eine höhere selbst zugeschriebene Sicherheit für alle Parameter bei männlichen gegenüber weiblichen Befragten sowie bei längerer gegenüber kürzerer Berufserfahrung, bei leitender Funktion gegenüber Fachärzten und Weiterbildungsassistenten sowie für chirurgisch gegenüber konservativ tätigen Ophthalmologen.

## Bedeutung der Gonioskopie

**Figure 4:**
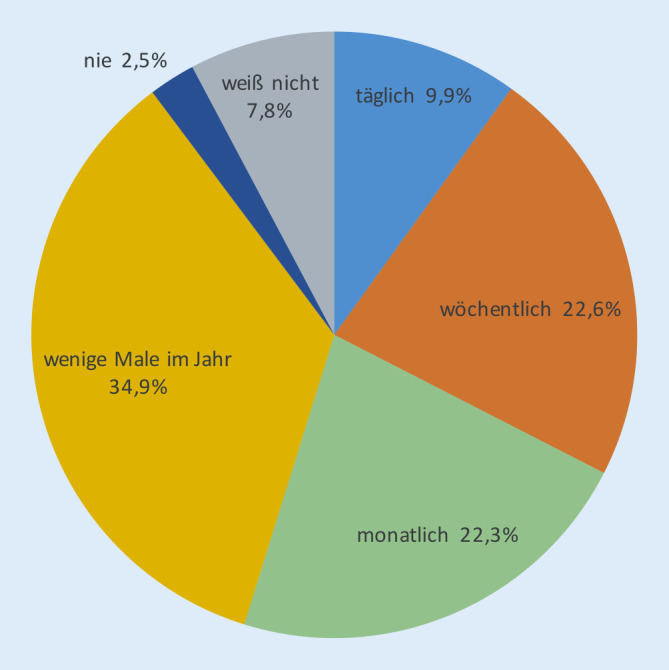

Gerade für die Kammerwinkelbeurteilung ergibt sich ein relativ hohes Maß an Unsicherheit – unter Weiterbildungsassistenten gestehen dies immerhin 38 % offen ein. Die Sicherheit und Unsicherheit der Gonioskopie sind auch im Zusammenhang zu betrachten mit der Häufigkeit der praktizierten Untersuchung des Kammerwinkels (Abb. 4).

Während etwa ein Drittel der Befragten täglich oder wöchentlich gonioskopiert, tut dies ein weiteres Drittel nur wenige Male im Jahr. Auffällig ist, dass Ärzte in leitender Position in Kliniken deutlich häufiger den Kammerwinkel untersuchen (63,0 % täglich oder wöchentlich) als Fachärzte (30,2 %) und Weiterbildungsassistenten (38,2 %).

## Versorgungspraxis

Die Gesichtsfelddiagnostik ist in der Praxis ein wichtiger Pfeiler der Glaukomversorgung – mehr als zwei Drittel der Befragten führen im ersten Behandlungsjahr zwei oder mehr Gesichtsfelduntersuchungen durch und anschließend etwa zur Hälfte einmalig und fast 40 % der Befragten zweimalig pro Jahr. Eine strukturelle Papillendarstellung führen etwa zwei Drittel der Augenärzte einmal im Jahr durch. Dabei gaben 75,8 % der Befragten an, eine OCT-Glaukomuntersuchung als sog. „individuelle Gesundheitsleistung“ (IGeL) anzubieten. Weitere 73,2 % boten eine Pachymetrie als IGeL an. Eine Konsultation von Augenkliniken für Zweitmeinungen erfolgt laut Angaben durchschnittlich bei etwa jedem 20. Glaukompatienten (Tab. 1).

**Table 1:** 

Gesichtsfelduntersuchung im 1. Jahr	1‑mal/Jahr	2‑mal/Jahr	3‑mal/Jahr	4‑mal/Jahr	Gar nicht	Weiß nicht/keine Angabe
	21,0 %	49,8 %	15,1 %	8,1 %	0,6 %	5,4 %
Gesichtsfelduntersuchung nach dem 1. Jahr	Maximal jedes 2. Jahr	1‑mal/Jahr	2‑mal/Jahr	3‑mal/Jahr	Nie	Weiß nicht/keine Angabe
	1,9 %	48,8 %	38,9 %	4,8 %	0,1 %	5,5 %
Strukturelle Papillendarstellung (OCT, HRT, Papillenfoto)	Maximal jedes 2. Jahr	1‑mal/Jahr	2‑mal/Jahr	3‑mal/Jahr	Nie	Weiß nicht/keine Angabe
	23,1 %	63,8 %	5,5 %	1,2 %	0,3 %	6,1 %
Überweisungen für Zweitbeurteilungen	≥ 10 % der Fälle	6–10 % der Fälle	2–5 % der Fälle	≤ 2 % der Fälle	Nie	Weiß nicht/keine Angabe
	5,6 %	13,4 %	34,3,%	30,2 %	6,9 %	9,6 %

## Rolle von Behandlungsleitlinien

Behandlungsleitlinien haben für die Befragten offenbar einen hohen Stellenwert für ihre Arbeit – so schätzen 44,7 % diese für „sehr hilfreich“ und weitere 47,6 % für „eher hilfreich“ ein. Dabei waren am bekanntesten die Leitlinien von DOG und BVA (93,1 %), gefolgt von denen der European Glaucoma Society (EGS – 58,4 %). Weniger bekannt hingegen waren die Guidelines der American Academy of Ophthalmology (AAO – 17,9 %).

## Offene Kommentare zur Glaukomversorgung

In einem offenen Kommentarfeld wurden die Befragten aufgefordert, eigene Angaben zu Problemen der Glaukomversorgung zu machen sowie konkrete Themenwünsche für die Aus- und Weiterbildung zu formulieren. Diese qualitativen Aussagen wurden nach Kategorien sortiert und illustrieren verschiedene Dimensionen und Schwerpunkte der jeweiligen Themenfelder.

### Kommentare zu konkreten Problemen der Glaukomversorgung

#### Diagnostik

- „Die Kammerwinkel-Diagnostik ist funktionell nur orientierend, und ich bin mir oft unsicher in dieser Überlegung.“
- „Eine exakte Papillenbeschreibung wird selbst von renommierten universitären Glaukomabteilungen nicht mehr praktiziert.“
- „Das gesamte Diagnostikkonzept muss neu sortiert werden.“
- „Im Alltag sind häufige Gesichtsfelduntersuchungen oft nicht realisierbar.“
- „Die langfristige Kontrolle der strukturellen und funktionellen Parameter sollte standardisiert werden.“
- „Zu jedem erhöhten Augendruck gehört eine Blutdruckmessung.“
- „Glaukome werden überdiagnostiziert und übertherapiert. Makropapillen und anatomische Normvarianten werden nicht erkannt.“

#### Aus- und Weiterbildung

- „Glaukombehandlung ist Fischen im Trüben. Wir brauchen klarere Behandlungsstandards und mehr Weiterbildung.“
- „Deutschlands Ophthalmologie ist Lichtjahre von einer standardisierten Ausbildung nach britischem/amerikanischem Vorbild entfernt.“
- „Die Forderung nach ‚evidenzbasierten‘ Studien wird der Geschwindigkeit des Fortschritts in der digitalen Sehnervenmessung nicht gerecht.“

#### Strukturelle Herausforderungen

- „Man sollte aufpassen, dass die Glaukomvorsorge nicht an Optiker abgegeben wird.“
- „Papillen-OCT und Makula-OCT sollte 1‑mal jährlich Kassenleistung sein sowie Tensiomessung für alle Patienten.“ (mehrfach genannt)
- „Die Erkennung eines Glaukoms gehört in die Grundversorgung.“
- „Die Kostenerstattung durch den Patienten ist besser als die Abrechnung der Nummer über die Kasse!“
- „Wünschenswert wäre, wenn die Fachgesellschaften sich dafür einsetzen würden, dass gute Aufklärung der Patienten entsprechend vergütet würde.“

### Themenwünsche für Leitlinien sowie für Aus-und Weiterbildung

- „Leitschemata zur einheitlichen Papillenbeurteilung“
- „Mehr Informationen über Auswertung von OCT bei einer Makro- und Mikropapille“
- „Schnell verfügbare, klare Fakten – kurze Zusammenfassungen für den schnellen Blick im Alltag“
- „Verständliche Hilfen zur Auswertung von Gesichtsfeldbefunden“
- „Darstellung von Fallstricken“
- „Mehr Leitlinien zu Normaldruckglaukomen“
- „Online-Video-Material zur Förderung der Adhärenz der Patienten“
- „Austauschplattform für schwerwiegende Fälle“
- „Weiterbildungskurse in den bildgebenden Methoden“
- „Bebilderte Fortbildungen zur Gonioskopie“
- „Regelmäßige Online-Schulungen, z. B. wie interpretiere ich ein Gesichtsfeld richtig?“
- „Weiterbildungen mit strikter Unabhängigkeit von Arzneimittelherstellern“
- „Weiterbildung auch bei Hausärzten und Internisten“

## Diskussion

Unsere Umfrage zur Glaukomversorgung in Deutschland zeigt einen Querschnitt über die praktizierten diagnostischen Verfahren. Die Ergebnisse offenbaren eine große Vielfalt, einzelne Kontroversen, aber auch überraschende Einigkeit der Auffassungen über Maß und Mittel der Diagnostik. Während zum einen die Beurteilung des Sehnervenkopfes an der Spaltlampe weiterhin als zentrales diagnostisches Instrument von den Befragten bestätigt wird, sticht daneben die hohe Bewertung der OCT-Glaukomdiagnostik hervor. Demnach sind neben dem klinisch-intuitiven Papillenbefund die Darstellung und Ausmessung des Sehnervenkopfes mittlerweile zu einem festen Bestandteil der Alltagsversorgung geworden.

## Grauzone Diagnosestellung

Die Unterscheidung zwischen „gesund“ und „krank“ ist gerade in der Glaukomdiagnostik schwierig. Bei der Frage der Treffsicherheit der Diagnose zwischen unterschiedlichen Untersuchern divergieren daher die Auffassungen unter den Befragten deutlich. Bekannt ist aus verschiedenen Untersuchungen zur Diagnosesicherung beim Glaukom, dass die Übereinstimmung verschiedener Untersucher („interobserver agreement“) höher ausfällt unter Glaukomspezialisten im Vergleich zu Nichtspezialisten [6, 11, 18], aber grundsätzlich auch niedriger als bei wiederholten Beurteilungen durch ein und denselben Untersucher („intraobserver agreement“) [1, 2, 13]. Eine Untersuchung in Mydriasis konnte zu einem höheren Konsens der Untersucher beitragen [7]. Weiterhin wurde beobachtet, dass die Übereinstimmung unter verschiedenen Untersuchern besser war anhand von Papillendarstellungen und deutlich schlechter bei Gesichtsfeldanalysen [4, 18]. In der Diagnostik der Papille stellte sich heraus, dass eine vergrößerte Cup-Disc-Ratio eher detektiert wurde als eine Verdünnung des neuroretinalen Randsaums [13] und die Verwendung einer zusätzlichen OCT-Untersuchung die diagnostische Genauigkeit der Diagnosestellung verbesserte [16]. In unserer Befragung deuten die Ergebnisse darauf hin, dass die Parallelität der verschiedenen, hoch bewerteten Untersuchungsparameter sowie engmaschige Kontrollintervalle als Mittel der Diagnosesicherung und der Verringerung von Beurteilungsfehlern verwendet werden. Für ausgewählte Fälle wird zudem eine aktive Überweisung für Zweitbeurteilungen gewählt.

## Assistierte statt automatisierte Diagnostik

Aktuell werden vielfach die Rolle von künstlicher Intelligenz und die Möglichkeiten sog. „Deep-Learning“-Algorithmen zur Verbesserung der Detektion pathologischer Prozesse in der Augenheilkunde diskutiert [1, 14]. In einem Cochrane-Review über verschiedene diagnostische Glaukomverfahren [8] konnte aus 106 Studien eine durchschnittliche Sensitivität (die Erfassung von „wirklich Kranken“ mit der jeweiligen Testmethode) von 70 % errechnet werden, worin sich die verschiedenen Verfahren (inklusive des OCT) kaum unterschieden. Wenn man sich diese Sensitivitätsquote der bisherigen Glaukomdiagnostik als Ausgangspunkt vor Augen führt, bedeutet dies jedoch, dass 30 % der reell Kranken damit übersehen werden („Falsch-Negative“). Bei jüngeren Erwachsenen unter 50 Jahren beträgt dieser Anteil sogar über 40 % [15]. Selbst wenn es gelänge, die Sensitivität mittels Deep-Learning-Verfahren tatsächlich etwas zu erhöhen [16, 17], ist zu erwarten, dass verfeinerte Instrumente der Papillendiagnostik in Zukunft weitere Additiva für die klinische Urteilsfindung sein werden, aber diese nicht ersetzen können.

Frühere Studien konnten belegen, dass die Sensitivität der binokulären Ophthalmoskopie der morphometrischen Diagnostik mittels OCT oder HRT nicht unterlegen war [3, 21]. Auch können die anamnestische Erfragung und die klinische Wahrnehmung der gesamtkörperlichen Situation nur durch einen unmittelbaren Kontakt mit dem Patienten erfolgen. Das eigene klinische Urteil in der Diagnosefindung bleibt demnach das Kernelement der Glaukomversorgung, das sich aus der Ophthalmoskopie und der Abwägung der verschiedenen messbaren diagnostischen Parameter und anamnestischer Angaben ergibt [22]. Es ist nicht auszuschließen, dass auch nichtmessbare, intuitive Faktoren bei dieser Urteilsbildung eine Rolle spielen, indem möglicherweise für besonders ängstliche oder multimorbide Patienten eher eine Glaukomdiagnose gestellt wird als für andere. Der Prozess der klinischen Urteilsfindung und das Zusammenspiel von vermeintlich „harten“ und „weichen“ Einflussfaktoren sollte daher Gegenstand weiterführender Forschung sein. Im besten Fall sollte der wachsende Einfluss der Bildgebung auch ein Ansporn sein, das eigene klinische Urteil umso kritischer zu fällen und zu hinterfragen.

Das heterogene Bild zur persönlichen Sicherheit in der Interpretation der verschiedenen Glaukomparameter ist auch ein Ausdruck für den jeweiligen klinischen Stellenwert. Hier ist eine Tendenz zu erkennen, dass der Papillenbeurteilung und -vermessung eine höhere und papillenferneren Parametern eine etwas geringere Rolle zugemessen wird. Gerade bei der Gonioskopie zeigt sich Unsicherheit vieler Befragter. Bekannt ist, dass die Untersuchung des Kammerwinkels mittels Kontaktglas unter den verschiedenen Glaukomparametern eher als wenig beliebt gilt und daher auch in anderen Teilen der Welt nicht standardmäßig durchgeführt wird [19]. Aus unseren Ergebnissen geht ebenfalls hervor, dass die Gonioskopie gerade von Ärzten in der Facharztausbildung, aber auch von Fachärzten relativ wenig praktiziert wird. Naheliegend ist, dass bei wenig Gebrauch auch nur eine geringere persönliche Sicherheit entsteht. Hier besteht die Gefahr einer zu großen Fokussierung auf einfacher zu erhebende morphometrische Parameter. Aus den offenen Kommentaren geht daher auch Kritik an einer zu geringen Standardisierung der Glaukomversorgung und der augenärztlichen Ausbildung hervor.

## Wünsche nach handhabbaren Behandlungsleitlinien

Unter den Ophthalmologen herrscht Einigkeit darin, dass Behandlungsleitlinien hilfreich und sinnvoll sind. Untersuchungen wie die Perimetrie oder strukturelle Papillendarstellungen werden nach der Auskunft der Befragten in angemessener Regelmäßigkeit wiederholt gemäß den Leitlinien von DOG/BVA [8, 9] oder der European Glaucoma Society (EGS) [10]. Zugleich wird von den Umfrageteilnehmern mehrfach der Wunsch nach einfachen und handhabbaren Hilfestellungen und unabhängigen Weiterbildungskursen geäußert. Hier könnten die bestehenden Leitlinien in Hinsicht auf ihre Praktikabilität im Behandlungsalltag überprüft und ggf. angepasst werden. Auch die hierzulande weniger bekannten Behandlungsrichtlinien („Preferred Practice Patterns“) [20] der American Academy of Ophthalmology (AAO) könnten als Vorbild dienen, die Standards für die Glaukomversorgung weiterzuentwickeln. Ein gemeinsamer Konsens der Fachgesellschaft über das notwendige Fachwissen zum Glaukom wäre sinnvoll, um noch gezielter Wissenslücken in Aus- und Weiterbildung zu adressieren und so die Sicherheit der Diagnostik zu verbessern.

Ein nicht unwesentlicher Teil der Glaukomversorgung betrifft den sog. Zweiten Gesundheitsmarkt jenseits der Erstattung durch die gesetzlichen Krankenkassen. Drei Viertel der Befragten geben an, OCT-Untersuchungen und Pachymetrien als individuelle Gesundheitsleistungen (IGeL) regelmäßig anzubieten. Hier stellt sich das gesundheitspolitische Problem, dass diese Untersuchungen als medizinischer Standard zwar in der Versorgungspraxis etabliert sind, aber dennoch privat den Patienten in Rechnung gestellt werden müssen. In den offenen Kommentaren zeigt sich ein geteiltes Bild hierzu: so wurde mehrfach der Wunsch geäußert, diese Untersuchungen (wie auch die Glaukomfrüherkennung) in den Leistungskatalog der gesetzlichen Krankenkassen zu überführen, während andere dies ablehnten. Hier stellen sich grundsätzliche gesundheitspolitische Fragen über den Stellenwert des „Gesundheitsproblems Glaukom“ in der Gesellschaft sowie darüber, ob und wie eine Finanzierung durch eine substanzielle Aufwertung der Glaukomversorgung angesichts der hohen Anschaffungskosten der diagnostischen Geräte gewährleistet werden kann.

Trotz einer sehr hohen Teilnehmerzahl unserer Umfrage ist die Rücklaufquote zur Anzahl der verschickten Einladungen per E‑Mail eher gering. Unsere Ergebnisse haben daher primär explorativen und weniger repräsentativen Charakter für die Gesamtheit aller Augenärzte und können die „wirkliche“ Versorgungspraxis nicht vollständig abbilden. So hat die Umfrage möglicherweise vornehmlich solche Ophthalmologen angesprochen, die ohnehin ein besonderes Interesse an der Glaukomversorgung haben. Eine weitere Schwierigkeit besteht auch darin, dass Selbstauskünfte zu einem sozial gewünschten Umfrageergebnis („social desirability bias“) neigen. Anzunehmen ist daher, dass die tatsächliche Versorgungspraxis – gerade auch in Bezug auf die Orientierung an Behandlungsleitlinien – in der Breite des Versorgungsgeschehens schlechter ausfällt als hier abgebildet. Zweifel und Unsicherheiten bei der Diagnosestellung haben wahrscheinlich eine noch höhere praktische Relevanz im Behandlungsalltag als hier abgebildet. Gerade die kritischen Kommentare und Wünsche für die Aus- und Weiterbildung zeigen Mängel auf und nennen konkrete Anstöße, wie die Glaukomversorgung in Zukunft weiter verbessert werden kann. Trotz aller Heterogenität der Versorgungslandschaft gilt es, die bestehenden Behandlungsstandards noch mehr publik zu machen, weiterzuentwickeln und im Alltag auch anzuwenden.

## Fazit für die Praxis

- Unter den verschiedenen Parametern der Glaukomdiagnostik erfährt die Papillenbeurteilung an der Spaltlampe die höchste Bewertung.
- Auch die OCT-Glaukomdiagnostik hat mittlerweile einen herausragenden Stellenwert und trägt zu einer assistierten – jedoch nicht automatisierten – Urteilsfindung bei.
- Papillenferne Parameter wie die Kammerwinkelbeurteilung werden tendenziell geringer eingestuft. Die Gonioskopie gehört für viele nicht zum Standardrepertoire der Diagnostik.
- Unsicherheiten in der Interpretation der verschiedenen Glaukomparameter bestehen. Mehr Behandlungsstandards und praxisnahe Hilfen für Aus- und Weiterbildung werden gewünscht.
- Die bestehenden Behandlungsleitlinien sollten mehr Anwendung im Versorgungsalltag finden. Ein gemeinsamer Konsens der Fachgesellschaft über das notwendige Fachwissen zum Glaukom wäre wünschenswert.
